# Improvement of Pyroelectric Cells for Thermal Energy Harvesting

**DOI:** 10.3390/s120100534

**Published:** 2012-01-05

**Authors:** Chun-Ching Hsiao, An-Shen Siao, Jing-Chih Ciou

**Affiliations:** Department of Mechanical Design Engineering, National Formosa University, No. 64, Wunhua Rd., Huwei Township, Yunlin County 632, Taiwan; E-Mails: qbasic147@gmail.com (A.-S.S.); opportunity8246@gmail.com (J.-C.C.)

**Keywords:** pyroelectricity, thermal energy, temperature variation rate, electrode

## Abstract

This study proposes trenching piezoelectric (PZT) material in a thicker PZT pyroelectric cell to improve the temperature variation rate to enhance the efficiency of thermal energy-harvesting conversion by pyroelectricity. A thicker pyroelectric cell is beneficial in generating electricity pyroelectrically, but it hinders rapid temperature variations. Therefore, the PZT sheet was fabricated to produce deeper trenches to cause lateral temperature gradients induced by the trenched electrode, enhancing the temperature variation rate under homogeneous heat irradiation. When the trenched electrode type with an electrode width of 200 μm and a cutting depth of 150 μm was used to fabricate a PZT pyroelectric cell with a 200 μm thick PZT sheet, the temperature variation rate was improved by about 55%. Therefore, the trenched electrode design did indeed enhance the temperature variation rate and the efficiency of pyroelectric energy converters.

## 1. Introduction

The task of harvesting energy from renewable sources such as thermal energy, light energy, wind power, vibration, or mechanical energy has stimulated important research efforts over the past years. Energy conversion devices, from millimeter scale down to microscale, have been presented with average powers in the 10 μW to 10 mW range. These devices for low grade waste heat have received significant attention due to the necessity to develop more energy efficient engineering systems. Therefore, an efficient energy conversion cell has become a crucial requirement in these applications.

Thermoelectric modules are the main means of harvesting energy from temperature gradients, and can generate electric output energies ranging from μW to kW. A temperature gradient (spatial gradient) leading to a heat flow through thermoelectric generators is converted into electric energy. Thermoelectric material properties are the key parameters in improving both the output power and the power efficiency. Small-scale thermoelectric generators are smaller than the temperature spatial fluctuation length, resulting in difficulties with temperature gradients. Usually, natural temperature time variations result in a thermal energy with an unstable temperature gradient, which is difficult to transform into electric energy using thermoelectric modules.

Contrary to thermoelectric generators, pyroelectric materials do not need a temperature gradient, but do require temporal temperature changes. The pyroelectric effect is mainly used for pyroelectric infrared temperature sensors. Thin-film pyroelectric sensors have many advantages, such as integration with on-chip circuitry, un-cooled detection, room-temperature operation, high speed, low system cost, portability and wide spectral response with high sensitivity. They have been successfully used in many applications, such as pollution monitoring, hot image detector devices, intruder alarms and gas analysis [1–4]. However, pyroelectricity has often been traditionally discarded as a useful energy source due to its low efficiency [5]. Nevertheless, pyroelectric materials have been used in the conversion of thermal energy to electric energy. Recently, one study focused on pyroelectric cells based on fabricated screen-printed PZT and commercial PVDF films proposed as thermal energy harvesting sources in order to supply low-power autonomous sensors [6]. It is possible to improve the design of pyroelectric converters by increasing the thickness of the PZT cells, decreasing the electrical capacitance of the PZT cells and by using PZT cells poled with a higher electrical field during the fabrication process.

Moreover, achieving an accurate efficiency of thermodynamic cycles has also been studied in <111> 0.75Pb (Mg_1/3_Nb_2/3_)-0.25PbTiO_3_ pyroelectric single crystals [7]. The conversion ratio for pyroelectric energy harvesting was much larger. In theory, it can reach the conversion ratio of the Carnot cycle, whatever the material properties. However, the conversion ratio of thermoelectric modules is highly limited by the material of fabrication’s properties. This current study was concerned with the design, assembly, and operation of a pyroelectric energy converter for harvesting waste heat for direct conversion into electricity by performing the Olsen cycle on co-polymer 60/40 P(VDF-TrFFE) thin films [8]. The largest energy density was 130 J/L at 0.061 Hz with temperature oscillations between 69.3 and 87.6 °C, while the largest power density was 10.7 W/L at 0.12 Hz between 70.5 and 85.3 °C. Therefore, pyroelectric energy conversion offers a novel and direct way to convert waste heat into electricity by alternatively heating and cooling a pyroelectric material, resulting in electricity.

The pyroelectric effect is the property of some dielectric materials with polar point symmetry which present a spontaneous electrical polarization as a function of temperature. Pyroelectric materials respond to changes in temperature which causes internal strain, and this in turn results in electrical charges on the material’s surface. The generated current of pyroelectric cells is based on the pyroelectric effect, converting temperature variation to a corresponding electrical output. The pyroelectric current is given by [1]:

(1) $$i_{p}=\text{dQ}/\text{dt}=\eta \times P\times A\times \text{dT}/\text{dt}$$

where η is the absorption coefficient of radiation, P is the pyroelectric coefficient of the pyroelectric materials, A is the electrode area, dT/dt is the temperature variation rate of the pyroelectric materials, Q is the induced charge and λ is the pyroelectric coefficient given by:

(2) $$\lambda ={\text{dP}}_{s}/\text{dT}$$

where P_s_ is the magnitude of the electrical polarization vector. Pyroelectric cells were sandwiched between top and bottom electrodes, as flat-plate capacitors, and poled along the axis perpendicular to the plates. P_s_ was perpendicular to the electrode surface while its magnitude equaled the electrode charge density. The thermal-isolation configuration, pyroelectric material properties, electrode layout and absorption coefficient of pyroelectric materials are the most important performance-enhancing qualities. From Equation (1), it can be seen that a higher temperature variation rate in pyroelectric cells leads to a higher response current in the devices. Moreover, a partially covered top electrode resulted in a higher responsivity than a fully covered electrode because it allowed the ZnO layer to make direct contact with the heat source [9].

Furthermore, a web-type top electrode has been designed to enhance the responsivity of ZnO pyroelectric sensors [10]. A larger area of the exposed ZnO layer led to more heat absorption and also to more dispersed top electrodes. Thus the web-type top electrode design was able to manage both the area of the exposed ZnO layer and the dispersion of top electrodes. The outer regions of the web-type electrode possessed a larger exposed ZnO layer area, while the inner regions possessed a lower dispersion of top electrodes. Therefore, the temperature variation rate increased in the pyroelectric films when a partially covered top electrode was applied. The partially covered electrode was extended to form a three-dimensional pattern on the responsive element of LiTaO_3_, with lateral temperature gradients induced on the sidewalls of the responsive element under homogeneous irradiation [11].

Thus, the temperature variation rate increased in the responsive element, and this then increased the responsivity of the pyroelectric cells. However, the temperature variation rate was difficult to extract from pyroelectric layers by experimental measurement. A finite element model was built using the commercial software package COMSOL to explore the temperature variation rate in a 200 μm thick PZT pyroelectric cell with a design of mesh electrode and cavities fabricated by wet etching PZT sheets for improving the energy conversion efficiency of PZT pyroelectric cells by pyroelectricity. The deeper cavities with a smaller electrode width had more potential to enhance the temperature variation rate in PZT pyroelectric sheets. However, PZT etchant had a low etching rate and isotropic etching, which resulted only an etching depth about 15 μm with a lower etching rate about 0.07 μm/min. Therefore, the etching process was deemed unsuitable to dig deeper cavities in PZT sheets [12].

In this paper, a finite element model built by the commercial multiphysics software COMSOL MULTIPHYSICS^®^ 3.5 was used to explore the temperature variation rate in commercial PZT pyroelectric cells. The electrode layouts and trenches in the PZT sheets were designed and implemented to enhance the temperature variation rate and the efficiency of pyroelectric harvesting converters. Then, improvement in temperature variation rates of the PZT pyroelectric cells was evaluated.

## 2. Materials and Methods

### 2.1. Simulation

A PZT pyroelectric cell with the dimensions of 18 mm × 18 mm × 0.214 mm was used. The cell comprised a 0.2 mm thick PZT sheet sandwiched between a top and bottom electrode. The electrodes were a 7 μm thick silver film. PZT samples were provided by ELECERAM TECHNOLOGY Co. A thick PZT sheet with a low electrical capacitance is more likely to generate higher voltages than a thin PZT film with a high electrical capacitance under a given temperature variation [6]. However, a thick pyroelectric material has a high thermal capacity, which hinders quick temperature variations. Obviously, a thin pyroelectric material has a high temperature variation rate. Although thin film deposition technology can be used to grow films with a lower thickness, pyroelectric properties usually decrease quickly. A commercial PZT bulk material possessing excellent pyroelectric properties together with a novel designed trenched electrode used to improve the temperature variation rate and enhance the efficiency of pyroelectric harvesting converters.

**Figure 1.**
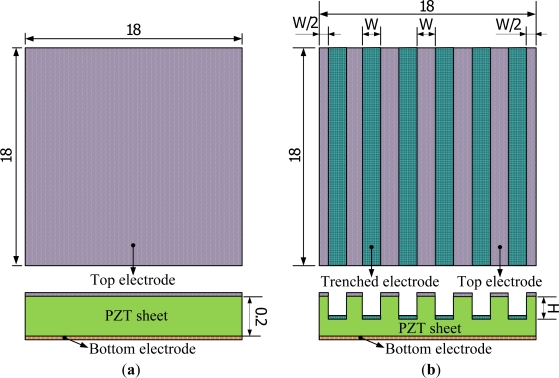
Top electrode dimensions in mm: (**a**) fully covered electrode type, (**b**) trenched electrode type.

A partially covered electrode has the potential to enhance the temperature variation rate in pyroelectric materials. Although increasing the bare electrode area can improve the heat absorption of pyroelectric materials and thus the temperature variation rate, the generated charges decrease due to the smaller top electrode area able to hold electrical charges. Inversely, increasing the top electrode area can increase the generated charges; the temperature variation rates decrease due to the heat absorption of pyroelectric materials blocked from the fully covered electrode. Therefore, the layout of the top electrode had the same width ratio as the top electrode and the trenched electrode to monitor both the temperature variation rate and the top electrode area. The profile of the trenches was rectangular in form, and was fabricated by a precision dicing saw (DS-150 II, EVERPRECISION TECH CO., LTD, Taiwan). The width of the trenches was controlled by that of a hub blade, and the depth of the trenches was manufactured and controlled by the dicing saw machine. The trenched electrode width size (W) was fixed at 200 μm, and the cutting depth (H) was simulated and varied from 50 to 150 μm. The trenched electrode was further deposited on the trenches to increase the electrode area as a way of improving pyroelectric currents, by an E-beam evaporator. Therefore, the area of top electrode in the fully covered and trenched electrode types was similar; approximately 324 mm^2^. The dimensions of the top electrodes are detailed in Figure 1.

**Figure 2.**
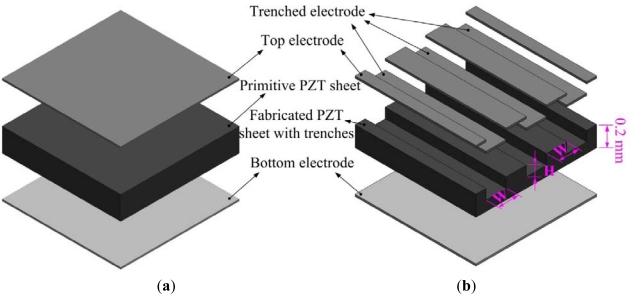
Exploded drawing of the PZT pyroelectric cells, (**a**) fully covered electrode type, and (**b**) trenched electrode type.

The cutting depth and the trenched electrode width seemed to be related to the thickness of PZT sheets, which could then be optimized by exploring and analyzing the temperature variation rate in the PZT pyroelectric cell. Figure 2 shows an exploded drawing of the PZT pyroelectric cells with various designs.

**Figure 3.**
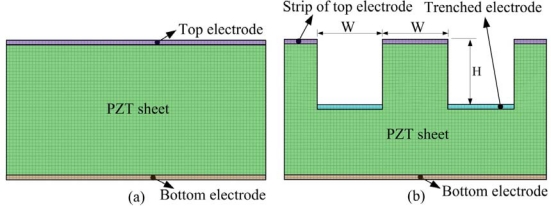
Finite element model for the PZT pyroelectric cells: (**a**) fully covered electrode type, (**b**) trenched electrode type.

A two-dimensional finite element model was generated by commercial multi-physics software COMSOL MULTIPHYSICS^®^ 3.5 to explore the temperature variation rate in a 200 μm thick PZT pyroelectric cell. The temperature variation rate was a dependent variable, and the trenched electrode width (W) and cutting depth (H), given variables. Material parameters of the PZT sheet and electrodes are listed in Table 1. All parameters were assumed to be isotropic. The models were meshed by regular mesh, as shown in Figure 3. The incident irradiation power applied to the top side of the PZT pyroelectric cells was approximately 1.228 × 10^−12^ W/μm^2^ [12]. Although the vertical sidewalls of trenches had a high potential to absorb the incidental irradiation power, there was no irradiation powers applied to make a worst-case scenario for probing the temperature fields and predicting the efficiency of PZT pyroelectric cells. Moreover, thermal isolation was applied to the rear side of the PZT pyroelectric cells, and the two lateral sides were symmetric, to serve as boundary conditions.

### 2.2. Fabrication Flow

**Figure 4.**
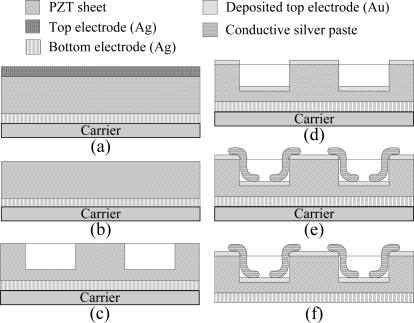
Process flow of the PZT pyroelectric cell: (**a**) a commercial PZT cell with dimensions of 18 mm × 18 mm × 0.214 mm attached to a carrier, (**b**) a wet etchant of HNO_3_: H_2_O = 7:3 used to remove the fully covered top electrode, (**c**) trenches with a width (W) and a depth (H) manufactured by a dicing saw machine, (**d**) a gold electrode deposited on the top side of the pyroelectric cells by an E-beam evaporator, (**e**) a silver paste used to connect the top and trenched electrodes, and (**f**) the carrier removed to finish the fabrication of PZT pyroelectric cells.

Properties of the commercial PZT pyroelectric cell are tabulated in Table 2. A tactic of trenching the PZT sheet was used to promote the temperature variation rate of the PZT pyroelectric cells. The process flow of the PZT pyroelectric cells is shown in Figure 4. Firstly, the PZT pyroelectric cell was attached to a glass substrate carrier, as shown in Figure 4(a). A wet etchant of HNO_3_-H_2_O = 7:3 was used to remove the fully covered top electrode, as shown in Figure 4(b). A precision dicing saw was used to manufacture the trenches with a width (W) and a depth (H) on the PZT sheet, as shown in Figure 4(c).

Although a PZT wet etchant could be used to make the trenches on the PZT sheets, a low etching rate and an isotropic etching prevented us from using wet etching. A gold film with a thickness of 100 nm was deposited on the top side of the PZT pyroelectric cells to finish the top and trenched electrodes using an E-beam evaporator, as shown in Figure 4(d). A lateral side of the PZT pyroelectric cells was daubed with a silver paste to connect the top and the trenched electrodes to increase the top electrode area and improve the generated charges, as shown in Figure 4(e). Finally, the carrier was removed to finish the fabrication of PZT pyroelectric cells, as shown in Figure 4(f).

## 3. Results and Discussion

Pyroelectric energy conversion offers a novel way to convert waste heat energy into electricity. Pyroelectric materials possess a spontaneous polarization in the absence of an applied electric field. The spontaneous polarization is strongly dependent on temperature due to the crystallographic structure of pyroelectric materials. When a PZT pyroelectric cell is subjected to temperature variation, its internal polarization produces an electrical field which induces voltage and current responses between the top and bottom electrodes. The responses are proportional to the temperature variation rate in the PZT pyroelectric materials. Although a thicker PZT pyroelectric sheet has a high potential to generate a high voltage response and possesses outstanding material properties, it possesses a larger heat capacity opposed to the temperature variation rate in PZT materials. The trenched electrode was designed to ameliorate the temperature variation rate in pyroelectric cells, and attempted to enhance the efficiency of pyroelectric harvesting converters.

**Figure 5.**
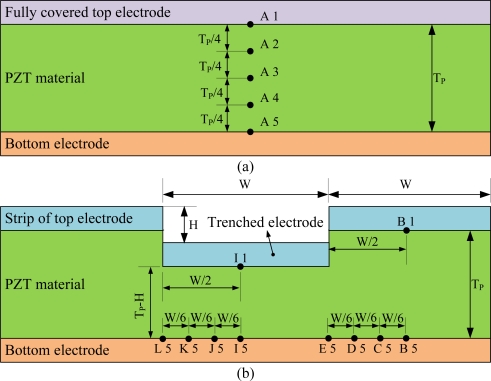
Points defined in the PZT sheets: (**a**) fully covered electrode type, (**b**) trenched electrode type.

Transient temperature fields in the PZT pyroelectric sheets were simulated. The points shown in Figure 5 were used to interpret the temperature variation rate in the PZT pyroelectric cells with the trenched electrode type, in comparison to the fully covered electrode type. A1 and B1 were defined at the top of the PZT sheet. A3 was defined at the middle of the PZT sheet. A5, B5, C5, D5 and E5 were defined at the bottom of the PZT sheet. Similarly, I1 was defined at the top of the PZT sheet under the trenched electrode. I5, J5, K5 and L5 were defined at the bottom of the PZT sheet under the trenched electrode.

**Figure 6.**
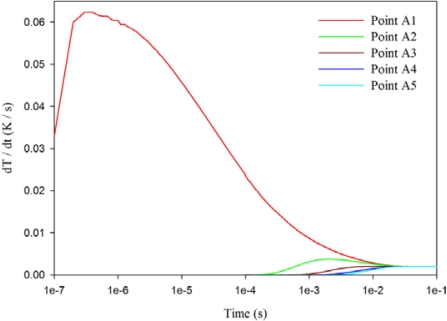
Relationship between temperature variation rate (dT/dt) and time in the PZT sheet at points A1–A5 for a 7 μm thick silver film of a fully covered top electrode.

Figure 6 shows the relationship between the temperature variation rate and time in the PZT pyroelectric cell at points A1 to A5, using a 7 μm thick silver film of the fully covered top electrode and a 200 μm thick PZT sheet. When the point approached the top side of the PZT sheet, the temperature variation rate increased gradually, and its maximum peak moved to the left, thereby reducing the response time. Hence, point A5 had the lowest temperature variation rate in the PZT sheet using the fully covered top electrode.

**Figure 7.**
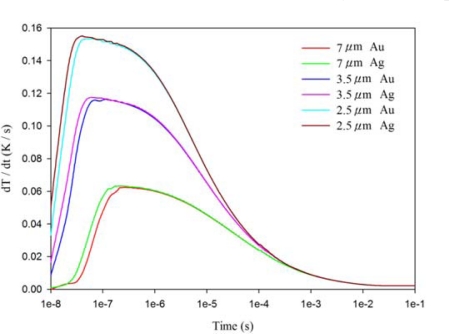
Relationship between temperature variation rate (dT/dt) and time in the PZT sheet at point A1 for various thicknesses and materials of fully covered top electrodes.

**Figure 8.**
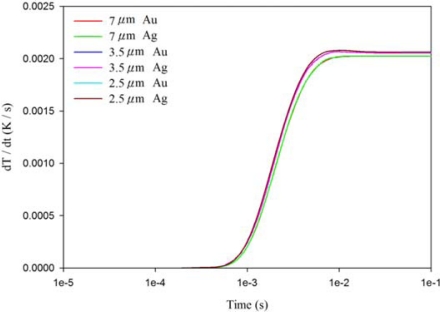
Relationship between temperature variation rate (dT/dt) and time in the PZT sheet at point A3 for various thicknesses and materials of fully covered top electrodes.

**Figure 9.**
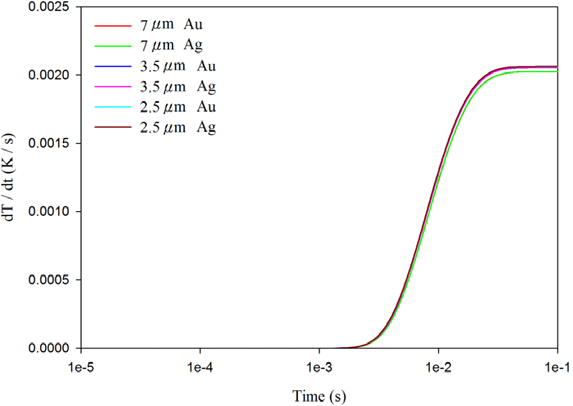
Relationship between temperature variation rate (dT/dt) and time in the PZT sheet at point A5 for various thicknesses and materials of fully covered top electrodes.

Figures 7–9 show the relationship between the temperature variation rate and time in a 200 μm thick PZT sheet at points A1, A3 and A5, using the fully covered electrode type under various materials and thicknesses of top electrode. When the point approached the bottom side of the PZT sheet, the temperature variation rate was not influenced by differing materials and thicknesses of the top electrode. However, the temperature variation rate at point A1 was greatly affected by changes in the thickness of the top electrode, but was not influenced by differing materials (gold or silver) of the top electrode. When the thickness of the silver electrode decreased by about 64% from 7 μm to 2.5 μm, the temperature variation rate at point A1 increased by around 146% from 0.0631 K/s to 0.1552 K/s. The response time of the maximum peak of the temperature variation rate at point A1 reduced from 240 ns to 40 ns, about 83%. Hence, a thinner top electrode had a more rapid response time and a higher temperature variation rate than a thicker top electrode. Therefore, a 100 nm thick gold film was deposited and used as the top electrode in the PZT pyroelectric cells to enhance the temperature variation rate in the PZT sheets.

**Figure 10.**
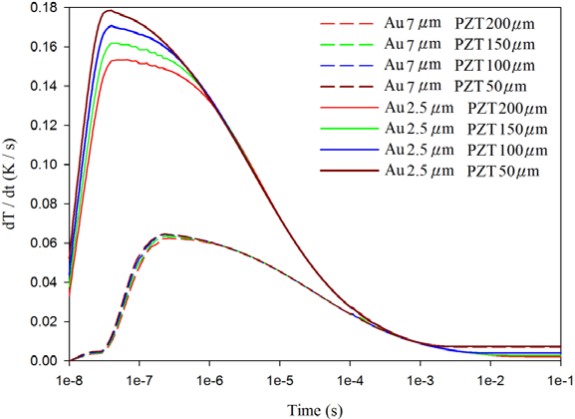
Relationship between temperature variation rate (dT/dt) and time in the PZT sheet at point A1 for various thicknesses of gold electrodes and PZT sheets.

**Figure 11.**
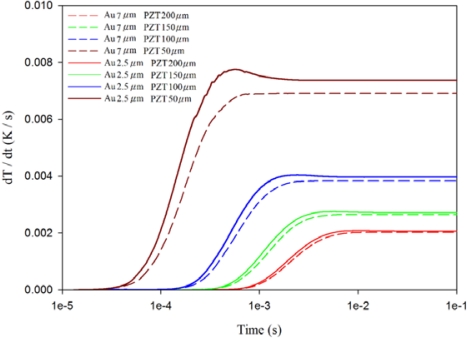
Relationship between temperature variation rate (dT/dt) and time in the PZT sheet at point A3 for various thicknesses of gold electrodes and PZT sheets.

**Figure 12.**
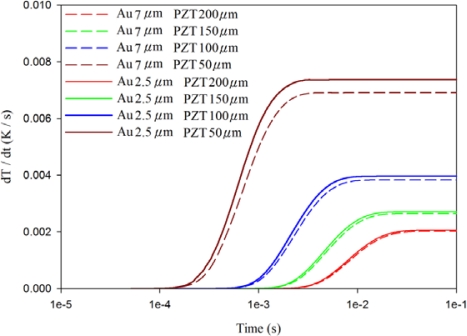
Relationship between temperature variation rate (dT/dt) and time in the PZT sheet at point A5 for various thicknesses of gold electrodes and PZT sheets.

Figures 10–12 show the relationship between temperature variation rate and time in various thicknesses of the PZT sheets at points A1, A3 and A5, with the fully covered electrode type under various thicknesses of gold electrodes. The temperature variation rate at the point approaching the top side of the PZT sheet was acutely affected by the thickness of the top electrode, and was also significantly influenced by the thickness of the PZT sheet when the thickness of the top electrode was reduced. When the thickness of the PZT sheet with a gold electrode of 2.5 μm was reduced by about 75% from 200 μm to 50 μm, the temperature variation rate at point A1 increased slightly from 0.15 K/s to 0.18 K/s, or about 20%.

Therefore, a decrease only in the thickness of the top electrode could improve the temperature variation rate at the point approaching the top side of the PZT sheet by reducing the thickness of the PZT sheet. Inversely, the temperature variation rate at the point approaching the bottom side of the PZT sheet was greatly affected by the thickness of the PZT sheet. The temperature variation rate at point A5 increased enormously from 0.002 K/s to 0.008 K/s—about 300%, when the thickness of the PZT sheet with a gold electrode of 2.5 μm was reduced by approximately 75% from 200 μm to 50 μm. Although the temperature variation rate at point A5 was the lowest, an increase in the temperature variation rate at the point approaching the bottom side of the PZT sheet could certainly improve the efficiency of PZT pyroelectric energy converters.

**Figure 13.**
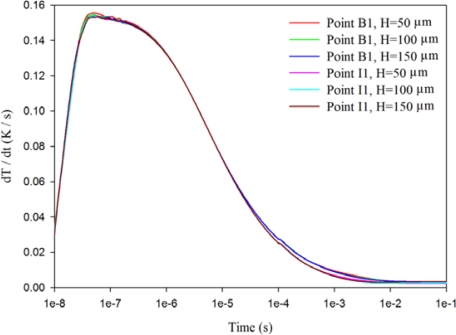
Relationship between temperature variation rate (dT/dt) and time in the PZT sheet at points B1 and I1 for various cutting depths under a gold film of 2.5 μm, an electrode width of 200 μm and a 200 μm thick PZT sheet.

**Figure 14.**
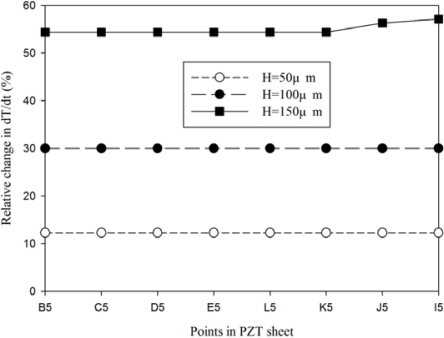
Relative change in the maximum peak value of the temperature variation rate at points B5, C5, D5, E5, I5, J5, K5 and L5 for the trenched electrode type, as compared to point A5 for the fully covered electrode type with various cutting depths.

When the trenched electrode type with a gold film of 2.5 μm and an electrode width of 200 μm and a 200 μm thick PZT sheet was used to fabricate the PZT pyroelectric cell, the temperature variation rate at point B1 showed little difference in comparison to the point I1 under various cutting depths, as shown in Figure 13. Figure 14 shows the relative change in the maximum peak value of the temperature variation rate at points B5, C5, D5, E5, I5, J5, K5 and L5 using the trenched electrode type, in comparison to point A5 using the fully covered electrode type under various cutting depths. The relative change in dT/dt can be expressed as:

(3) $$\text{dT}/\text{dt}\;(\%)=\left[{\left(\text{dT}/\text{dt}\right)}_{\text{trench}}-{\left(\text{dT}/\text{dt}\right)}_{\text{primitive}}\right]/{\left(\text{dT}/\text{dt}\right)}_{\text{primitive}}\times 100\%$$

where (dT/dt)_trench_ is the maximum peak value of the temperature variation rate at points B5, C5, D5, E5, I5, J5, K5 and L5 for the trenched electrode type, and (dT/dt)_primitive_ is the maximum peak value of the temperature variation rate at point A5 for the fully covered electrode type. It showed that the temperature variation rate at the bottom side of the PZT sheet promoted a deepening of the cutting depth.

**Figure 15.**
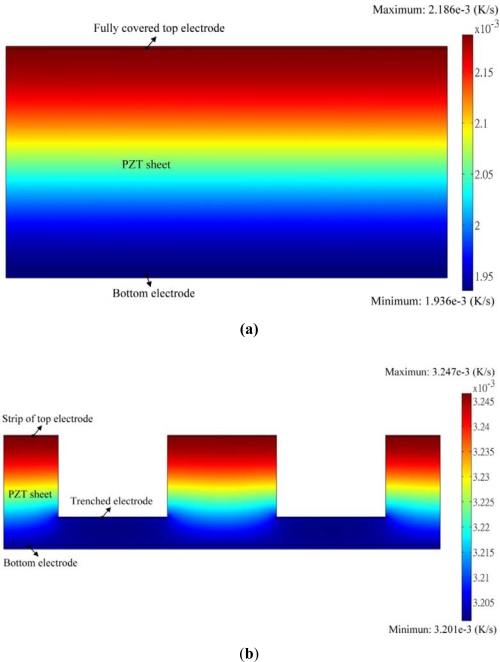
Transient temperature variation field in the PZT pyroelectric cells: (**a**) fully covered electrode type, (**b**) trenched electrode type.

Figure 15(a) shows the transient temperature variation field at the maximum peak’s time at point A5 when the fully covered electrode type with a gold film of 2.5 μm was used to fabricate the PZT pyroelectric cell.

The temperature variation rate in the PZT sheet increased gradually toward the top electrode as a result of the incidental radiation power applied to the top electrode. Figure 15(b) shows the transient temperature variation field at the maximum peak’s time at point B5 when the trenched electrode type with a gold film of 2.5 μm, an electrode width of 200 μm and a cutting depth of 150 μm was used to fabricate the PZT pyroelectric cell. When the cutting depth was about 150 μm, the temperature variation rate at the bottom side of the PZT sheet was improved by about 55%. Hence, pyroelectric cells with the trenched electrode design could efficiently enhance the temperature variation rate in pyroelectric materials due to lateral temperature gradients induced by the trenched electrode. The trenched electrode did indeed improve the temperature variation rate, and it further meliorated the efficiency of the pyroelectric harvesting converters.

## 4. Conclusions

Although a PZT pyroelectric cell with a thicker PZT sheet has high potential in generating electricity pyroelectrically, it possesses a larger heat capacity that opposes the temperature variation rate in PZT materials. Therefore, we anticipated that trenching the PZT sheets to produce deeper trenches could effectively enhance the temperature variation rate in a thicker PZT pyroelectric cell as a result of lateral temperature gradients induced by the trenched electrode. The design of the trenched electrode did indeed improve the temperature variation rate in pyroelectric cells and the efficiency of the pyroelectric harvesting converters.

## Figures and Tables

**Table 1.** Material parameters used for finite element analysis.

**Material**	**Thermal conductivity (Wm^−1^K^−1^)**	**Specific heat (Jg^−1^K^−1^)**	**Density (gcm^−3^)**	**Thickness (μm)**
**Silver electrode**	429	0.235	10.53	2.5–7
**Golden electrode**	317	0.129	19.3	2.5–7
**PZT**	2.1	0.36	7.97	200

**Table 2.** Properties of the commercial PZT pyroelectric cells.

**Sample ID**	**Thickness (μm)**	**Area (mm^2^)**	**Size (mm × mm)**	**Relative dielectric constant (ε_33_^T^/ε_O_)**	**Density (g/cm^3^)**	**Poling field (V/μm)**
KA	200	324	18 × 18	2,100	7.9	3.5
